# Most published meta-regression analyses based on aggregate data suffer from methodological pitfalls: a meta-epidemiological study

**DOI:** 10.1186/s12874-021-01310-0

**Published:** 2021-06-15

**Authors:** Michael Geissbühler, Cesar A. Hincapié, Soheila Aghlmandi, Marcel Zwahlen, Peter Jüni, Bruno R. da Costa

**Affiliations:** 1 Institute of Social and Preventive Medicine (ISPM), University of Bern, Bern, Switzerland; 2 Institute of Primary Health Care (BIHAM), University of Bern, Bern, Switzerland; 3 Department of Chiropractic Medicine, Faculty of Medicine, Balgrist University Hospital and University of Zurich, Zurich, Switzerland; 4 Epidemiology, Biostatistics and Prevention Institute (EBPI), University of Zurich, Zurich, Switzerland; 5 Applied Health Research Centre (AHRC), Li Ka Shing Knowledge Institute of St. Michael’s Hospital, Toronto, Canada; 6 Basel Institute for Clinical Epidemiology and Biostatistics, University Hospital Basel, Basel, Switzerland; 7 Department of Medicine, University of Toronto, Toronto, Canada; 8 Institute of Health Policy, Management and Evaluation (IHPME), Dalla Lana School of Public Health, University of Toronto, Toronto, Canada

**Keywords:** Meta-regression, Methodological pitfalls, Meta-analysis, Epidemiologic methods

## Abstract

**Background:**

Due to clinical and methodological diversity, clinical studies included in meta-analyses often differ in ways that lead to differences in treatment effects across studies. Meta-regression analysis is generally recommended to explore associations between study-level characteristics and treatment effect, however, three key pitfalls of meta-regression may lead to invalid conclusions. Our aims were to determine the frequency of these three pitfalls of meta-regression analyses, examine characteristics associated with the occurrence of these pitfalls, and explore changes between 2002 and 2012.

**Methods:**

A meta-epidemiological study of studies including aggregate data meta-regression analysis in the years 2002 and 2012. We assessed the prevalence of meta-regression analyses with at least 1 of 3 pitfalls: ecological fallacy, overfitting, and inappropriate methods to regress treatment effects against the risk of the analysed outcome. We used logistic regression to investigate study characteristics associated with pitfalls and examined differences between 2002 and 2012.

**Results:**

Our search yielded 580 studies with meta-analyses, of which 81 included meta-regression analyses with aggregated data. 57 meta-regression analyses were found to contain at least one pitfall (70%): 53 were susceptible to ecological fallacy (65%), 14 had a risk of overfitting (17%), and 5 inappropriately regressed treatment effects against the risk of the analysed outcome (6%). We found no difference in the prevalence of meta-regression analyses with methodological pitfalls between 2002 and 2012, nor any study-level characteristic that was clearly associated with the occurrence of any of the pitfalls.

**Conclusion:**

The majority of meta-regression analyses based on aggregate data contain methodological pitfalls that may result in misleading findings.

**Supplementary Information:**

The online version contains supplementary material available at 10.1186/s12874-021-01310-0.

## Background

Due to clinical and methodological diversity, clinical studies included in meta-analyses often differ in ways that lead to differences in treatment effects across studies [1]. Thus, a simple pooled effect size generally does not solely reflect the treatment effect on the outcome of interest, but also the effects of clinical or methodological characteristics that are unequally distributed across studies, and thus, potential effect modifiers. The assessment of covariates that are potentially associated with treatment effect is generally recommended, [2, 3] using meta-regression to explore associations between study-level characteristics and treatment effect [3]. However, three key pitfalls of meta-regression, if overlooked or ignored, may lead to invalid conclusions.

First, in meta-regression on aggregate data, associations between average patient characteristics and the pooled treatment effect do not necessarily reflect true associations between the individual patient-level characteristics and treatment effect [4, 5]. The difference between associations of treatment effects with average patient characteristics at group level and true associations with individual patient level characteristics has been referred to as ecological fallacy or aggregation bias [4]. It reflects a logical fallacy in the interpretation of observed data, and findings at the group level may be either an under- or overestimation of the real association between patient-level characteristics and treatment effect. Second, meta-regression models can be overfitted if the number of studies per examined covariate is low. A consequence of overfitting can be spurious associations between covariates and treatment effect due to idiosyncrasies of the data [6]. The latest version of the Cochrane Handbook suggests a minimum of 10 studies per examined covariate in meta-regression, [3] analogous to the traditional rule of thumb used to minimize the risk of overfitting in logistic and Cox regression models of at least 10 events per included covariate [7]. However, a recent study suggested that the number of observations required per covariate in ordinary least-squares linear regression may be considerably lower [6], and it remains unclear whether this also partially applies to the case of weighted random-effects meta-regression. Third, meta-regression analyses that regress treatment effects against the risk of the analysed outcome observed in included trials are difficult to interpret as the observed risk included as a covariate is also incorporated in the expression of the treatment effect used as the dependent variable in meta-regression. Regression to the mean will therefore result in an inherent correlation between covariate and dependent variable in meta-regression. In the extreme case of the true risk ratio or odds ratio of every trial being equal to 1, the introduced correlation can be as pronounced as -0.71, [8] even though covariate and treatment effect are truly unrelated [4, 9]. Methods to overcome this problem have been published, but are rarely used [10–13]. The primary objectives of this meta-epidemiological study were therefore, to determine the frequency of these three pitfalls in meta-regression analyses published in the medical literature. Due to limited resources and based on the publication timing of most methodological literature on meta-regression analysis, we limited our focus to the years 2002 and 2012. The secondary objectives were to examine associations between characteristics of journals, authors and methods with the occurrence of these pitfalls, and explore changes over time between 2002 and 2012.

## Methods

### Data source

We searched Medline through PubMed using the following search terms: *meta-analysis* OR *systematic[sb],* as used in previous meta-epidemiological studies [14, 15] to search for systematic reviews and meta-analyses. As the use of meta-regression analysis is not always reported in the title and abstract of publications, we first identified meta-analyses in the published medical literature and then screened their full-text for meta-regression analyses. We limited our search to the PubMed entry years 2002 and 2012. We chose 2002 as the comparator year for 2012 because most methodological articles addressing issues in meta-regression analyses [4, 5, 9, 16] had been published before 2002. The search for 2002 reports was done in June 2012; the search for 2012 reports was done in June 2013 and updated in January 2014. Based on a computer-generated sequence of random numbers, we identified random samples of reports from 2002 and 2012.

### Study selection

Eligible were all aggregate-level meta-analyses published in 2002 and 2012 in which at least one study was a randomised or quasi-randomised controlled trial, which reported on a meta-regression analysis [4] examining the association between one or several covariates and the estimated pooled treatment effect on a clinical outcome. Meta-regression analyses were considered irrespective of whether they related to the primary or secondary outcome variables. We excluded meta-regression analyses that did not have between-group comparisons (i.e., treatment effect estimates comparing two trial arms on a specific outcome) as the dependent variable, and meta-regression analyses for which results were not reported. During the first phase, one reviewer (MG) identified all aggregate-level meta-analyses based on titles and abstracts, excluding guidelines, network meta-analyses, and meta-analyses of individual patient data. A second reviewer (BdC) screened a randomly selected sample of 200 citations for each year. Percent agreement between the two screeners was 88% (chance-corrected agreement: kappa, 0.68). During a second phase, one reviewer (MG) determined eligibility based on full texts of all aggregate-level meta-analyses, and of all citations, for which it was unclear whether they reported on an aggregate-level meta-analysis.

### Data extraction

A standardised and piloted data extraction form accompanied by a codebook was used for data extraction. We determined whether meta-regression analyses included at least one of three previously described pitfalls of meta-regression: (1) ecological fallacy, [4, 5] (2) a high risk of overfitting (conceptualized as a meta-regression with less than 5 studies per covariate) [3, 6, 7] and (3) inappropriate methods to regress treatment effects (assessed with binary or continuous outcomes) against the risk of the analysed outcome observed in included studies [10–13]. We classified meta-regression analysis as susceptible to ecological fallacy if one or more of the covariates included in the analysis was an average estimate of a patient level characteristic such as mean age or proportion of females. We also examined whether authors of published reports recognized the limitations associated with these pitfalls if their meta-regression analysis was susceptible to them. The extraction of these data was done independently and in duplicate (MG, SA). Any disagreements were resolved by discussion, and consultation with a third author (BdC), if needed.

Additional data extraction, done by a single author (MG), involved extraction of the following characteristics: the name of the journal and journal characteristics (impact factor, type of journal, and appearance on the list of core clinical journals on PubMed), affiliation of the authors with industry, affiliation of the authors with an institute with statistical expertise (mainly biostatistics or epidemiology department), the number of studies included in the review, design of the studies included in the review and in the meta-regression analysis, whether the outcome was continuous or binary, the medical field of speciality, and the category of therapy (e.g., drugs, surgery, etc.). For all reports, we used the impact factor in the year 2007 (midpoint between 2002 and 2012). Our operationalization of these variables is reported in Additional file 1: Tables A-C.

### Analysis

Our sample of 2,404 records per entry year (4,808 total) was based on a pilot study of a random sample of 50 records from 2012, in which we identified 20 (40%) meta-analyses, 3 of which included a meta-regression analysis (6.0%, 95% confidence interval [CI] 2.1 to 16.2%). Assuming that the yield of meta-regression analyses in 2002 would be about one third of the yield in 2012, we estimated that the yield of meta-regression analyses in the total random sample of 4,808 citations would be 192 (95% uncertainty interval, 66 to 519). Our target sample size of 192 would yield 95% confidence intervals ranging from 14 to 26% if the prevalence of a potential pitfall was 20%, and ranging from 43 to 57% if the prevalence of a potential pitfall was 50%.

The primary outcome was the proportion of meta-analyses with at least one meta-regression analysis that was potentially influenced by one of the 3 assessed methodological pitfalls in our random samples for the years 2002 and 2012. Secondary outcomes were the proportion of meta-analyses with at least one meta-regression analysis with ecological fallacy; the proportion of meta-analyses with at least one meta-regression analysis with a high risk of overfitting; the proportion of meta-analyses with at least one meta-regression analysis with inappropriate methods to regress treatment effects against the risk of the analysed outcome observed in included studies; and the proportion of meta-analyses with at least one meta-regression analysis that recognized the limitations when these pitfalls were present. We used Firth’s logistic regression to investigate the association between study characteristics and the occurrence of pitfalls, using one predictor at a time. Firth’s logistic regression model uses a penalized maximum likelihood that produces more accurate inference in small samples than the standard maximum likelihood based logistic regression estimator, and achieves convergence in the presence of separation with better coverage probability [17, 18]. Odds ratios (ORs) and 95% CIs were derived from the models, with ORs above 1 indicating that the study characteristic was positively associated with a pitfall, and ORs below 1 indicating a negative association. All analyses were conducted in STATA 13, and 95% CIs were two-sided.

## Results

**Fig. 1:**
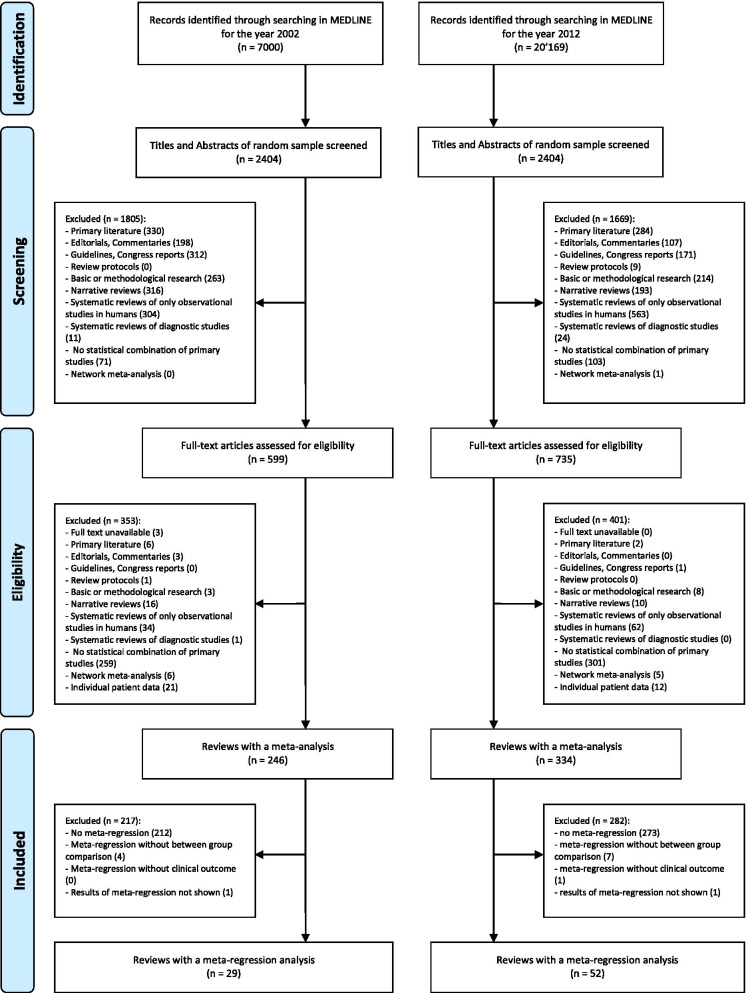
Flow diagram of the study selection process

Our search of 2002 and 2012 reports resulted in 7,000 citations for 2002 and 20,169 citations for 2012 (Fig. 1). Out of the random samples of 2,404 reports for each year, 599 in 2002 and 735 in 2012 were considered to be potentially eligible based on their title and abstract. Full-text analysis of these yielded 246 and 334 published reports describing aggregate-level meta-analyses that included at least one randomized trial in 2002 and 2012, respectively. 29 and 52 reports published in 2002 and 2012, respectively, were deemed to meet our eligibility criteria (81 meta-analyses with at least one meta-regression analysis in total).

Table 1 presents characteristics of included meta-analyses with meta-regression by year. Overall, the 81 meta-analyses included a median of 23 studies (IQR 13 to 41 studies), with 73 of these (90%) including ≥ 10 studies. The median journal impact factor was 4.2 (IQR 2.3 to 6.1). 15 meta-analyses (19%) were published in a general medical journal, and 19 meta-analyses (23%) in one of PubMed’s core clinical journals. 38 meta-analyses (47%) examined drug interventions, and 48 (59%) used a continuous primary outcome measure. The most common medical fields represented were psychiatry and psychology (16%), cardiology (12%) and oncology (10%), with a wide range of fields making use of meta-regression analyses (Table 1). Five meta-analyses (6%) had authors affiliated with industry, while 35 (43%) had authors affiliated with a biostatistics or epidemiology department.

**Table 1:** Characteristics of included studies with meta-regression analysis

Characteristics	Total (*N* = 81)	Year 2002 (*N* = 29)	Year 2012 (*N* = 52)
Journal characteristics			
Journal Impact Factor, median (IQR)	4.2 (2.3–6.1)	4.4 (2.9–5.6)	3.9 (2.0–6.6)
General medical journal	15 (19%)	5 (17%)	10 (19%)
Core clinical journals	19 (23%)	9 (31%)	10 (19%)
Author characteristics			
Affiliated with industry	5 (6%)	3 (10%)	2 (4%)
Affiliated with biostatistics or epidemiology department	35 (43%)	16 (55%)	19 (37%)
Ten or more of studies	73 (90%)	26 (90%)	47 (90%)
Drug intervention	38 (47%)	15 (52%)	23 (44%)
Type of outcome in meta-regression analysis			
Binary	39 (48%)	13 (45%)	26 (50%)
Continuous	48 (59%)	18 (62%)	30 (58%)
Clinical field			
Psychiatry & Psychology	13 (16%)	5 (17%)	8 (15%)
Cardiology	10 (12%)	3 (10%)	7 (13%)
Oncology	8 (10%)	1 (3%)	7 (13%)
Infectious disease	7 (9%)	2 (7%)	5 (10%)
Endocrinology & Metabolism	7 (9%)	3 (10%)	4 (8%)
Surgery	6 (7%)	0 (0%)	6 (12%)
General internal medicine	5 (6%)	3 (10%)	2 (4%)
Paediatrics	4 (5%)	2 (7%)	2 (4%)
Nutrition & dietetics	4 (5%)	0 (0%)	4 (8%)
Rheumatology	4 (5%)	3 (10%)	1 (2%)
Miscellaneous	13 (16%)	7 (24%)	6 (12%)

There were no important differences in baseline characteristics between the two assessed years

### Main outcome

Table 2 summarizes the prevalence of the three key pitfalls of meta-regression-analyses in the years 2002 and 2012. Overall, we found at least one of the assessed pitfalls in 70% of included reports (57/81; 95% CI 60% to 79%) with similar numbers in 2002 (72%, 95% CI 54% to 85%) and 2012 (69%, 95% CI 56% to 80%). Ecological fallacy was the most common issue, observed in 20 reports in 2002 (69%, 95% CI 51% to 83%) and 33 in 2012 (63%, 95% CI 50% to 75%). A high risk of overfitting was found in 6 reports in 2002 (21%, 95% CI 10% to 38%) and 8 in 2012 (15%, 95% CI 8% to 28%). Inappropriate methods to regress treatment effects against the risk of the analysed outcome were described in 2 reports in 2002 (7%, 95% CI 2% to 22%) and 3 in 2012 (6%, 95% CI 2% to 16%). Only two out of the 57 meta-analyses with at least one problematic meta-regression analysis (4%) provided a frank discussion of the limitations of meta-regression; in both, average patient characteristics were regressed against treatment effects, and the limitations of this approach were addressed in the discussion section [19, 20].

**Table 2:** Prevalence estimates with 95% confidence intervals of any potential pitfalls in meta-regression-analyses

Pitfall	Total (*N* = 81)	Year 2002 (*N* = 29)	Year 2012 (*N* = 52)
Ecological fallacy	53 (65%, 55 to 75%)	20 (69%, 51 to 83%)	33 (63%, 50 to 75%)
Overfitting	14 (17%, 11 to 27%)	6 (21%, 10 to 38%)	8 (15%, 8 to 28%)
Meta-regression on risk of the analysed outcome	5 (6%, 3 to 14%)	2 (7%, 2 to 22%)	3 (6%, 2 to 16%)
Any potential meta-regression pitfall	57 (70%, 60 to 79%)	21 (72%, 54 to 85%)	36 (69%, 56 to 80%)

### Characteristics associated with pitfalls

In logistic regression analyses, our results were most compatible with no important associations between publication year, journal characteristics, affiliation of study authors, the number of studies included in the review, whether the outcome was continuous or binary, category of therapy, and a composite of any of the three pitfalls (Table 3), or each of the three pitfalls separately (Additional file 1: Table C).

**Table 3:** Association between any inappropriate meta-regression and review characteristics

Any potential meta-regression pitfall	Yes (n = 57)	No (n = 24)	Odds Ratio (95% CI)
Published in 2012	36 (63%)	16 (67%)	0.87 (0.33 to 2.34)
Journal characteristics			
Core clinical journals	12 (21%)	7 (29%)	0.64 (0.22 to 1.85)
General medical journals	11 (19%)	4 (17%)	1.13 (0.34 to 3.77)
Impact factor higher than median	29 (51%)	11 (46%)	1.22 (0.47 to 3.11)
Author characteristics			
Affiliated with industry	5 (9%)	0 (0%)	5.13 (0.27 to 96.57)
Affiliated with biostatistics or epidemiology department	23 (40%)	12 (50%)	0.68 (0.27 to 1.75)
Ten or more of studies	53 (93%)	20 (83%)	2.61 (0.64 to 10.61)
Drug intervention	28 (49%)	10 (42%)	1.33 (0.52 to 3.44)
Binary outcome variable	25 (44%)	14 (58%)	0.57 (0.22 to 1.47)

Odds ratios are for the comparison of meta-regression analyses with the characteristic as compared to meta-regression analyses without the characteristic. An odds ratio of 2.61 for ‘Ten or more studies’ indicates, for example, that the odds of any potential meta-regression pitfall is 2.61 times higher in meta-regression analyses that include 10 or more studies as compared with meta-regression analyses that include a lower number of studies

## Discussion

In this meta-epidemiological study based on a random sample of 4,808 reports published in 2002 and 2012, we found 580 aggregate-level meta-analyses, of which 81 included at least one meta-regression analysis. Of these 81 meta-analyses, 57 (70%) were affected by at least one of three key pitfalls of meta-regression analyses addressed in our study—ecological fallacy, overfitting, or inappropriate methods to regress treatment effects against the risk of the analysed outcome. This suggests that about one out of ten meta-analyses are potentially flawed and may report invalid findings. In only two of the 57 meta-regression analyses with pitfalls, did authors explicitly acknowledge the limitations of their findings derived from meta-regression analysis. We found little evidence for associations between inappropriate meta-regression and characteristics of meta-analyses. There was inconclusive evidence with regard to the association between journal, author, and characteristics of meta-analyses and the odds of pitfalls of meta-regression. The negative association between the prevalence of pitfalls in meta-regression analyses and authors affiliated with biostatistics or epidemiology department (OR 0.68, 95% CI 0.27 to 1.75), although imprecise, is noteworthy. In addition, authors with such affiliation were less frequent in analyses published in 2012 (37%) than in 2002 (55%). This is striking and may reflect an increase in the availability of software for non-statisticians to conduct meta-regression analysis. This underlines the importance of a collaboration with a statistician experienced in evidence synthesis when conducting meta-regression analyses [21]. In addition, we did not find evidence for a difference when comparing the years 2002 and 2012.

### Strengths and limitations

Our meta-epidemiological study has several strengths. First, we used a systematic and broad search strategy using a validated filter to find systematic reviews and meta-analyses. We refrained from using filters specific to meta-regression, as many meta-regression analyses were not mentioned in titles and abstracts of eligible meta-analyses. Second, our use of two random samples of meta-analyses from 2002 and 2012 suggests generalizability of our findings to published meta-analyses with meta-regression indexed in PubMed. Third, we conducted a sample size consideration prior to our screening, which was recently proposed by Giraudeau et al. [22] who also provide a relevant framework for this purpose. In addition, data extraction was done using a dedicated data extraction form, and carried out partly in duplicate with discrepancies resolved through discussion and consensus, which minimized data extraction errors.

Our study has limitations. First, the number of meta-analyses with meta-regression analysis identified in our random samples was only 81, and therefore at the lower end of what we had expected based on our sample size consideration, which in turn has limited our statistical power to detect associations between pre-specified study characteristics and pitfalls. Second, our findings on overfitting may underestimate the true frequency of this pitfall as the threshold of less than 5 trials per covariate was more conservative than currently suggested in the Cochrane Handbook [3]. Third, we only considered meta-regression analyses of clinical studies on treatment effects, but the same principles and pitfalls also apply to meta-analyses of studies with other purposes and designs [4]. Forth, the most recent studies included in our analyses were published in 2012. Fifth, we did not investigate whether meta-regression analyses were pre-specified in the protocol. It is important that meta-regression analyses are pre-specified with as much detail as possible in a review protocol to reduce the risk of false-positive conclusions [4]. With the ever-growing increase in publications of review protocols, this would be a feasible and important issue to be investigated in a future methodological study of meta-regression analyses.

### Context

To our knowledge, no prior meta-epidemiological study has quantified the number of meta-regression analyses affected by these pitfalls. The problem of ecological fallacy in meta-regression analysis is well known [4, 5, 23, 24]. For example, Berlin and colleagues [5] showed that meta-regression based on aggregate data failed to detect an interaction between allograft failure after anti-lymphocyte antibody induction therapy and elevated panel reactive antibodies in patients after renal transplantation, whereas an analysis of individual patient data showed evidence for such an interaction. The high prevalence of meta-analyses with meta-regression that are subject to the ecological fallacy in both 2002 and 2012 suggests that published recommendations have had limited impact [2, 4, 25]. Common covariates prone to ecological bias were age, gender or baseline value of the outcome variable. Valid investigations of interactions between treatment effect and patient-level characteristics require the analysis of individual patient data in at least some of the trials included in a meta-analysis [26]. When some trials have individual patient data available, a Bayesian meta-regression approach combining associations derived from individual and aggregate level data can be used [27]. This method first quantifies the association between patient characteristics and treatment effect for each type of data separately. It then tests whether the association estimated based on individual patient data is different from the association based on aggregate level data. If the test indicates no difference above and beyond chance, the associations can then be combined using appropriate weighting.

The minimum number of trials per covariate in meta-regression analyses required to minimize the risk of overfitting is unknown. The Cochrane Handbook suggest a minimum of 10 studies per examined covariate, [3] but a recent study suggested a considerably lower number of observations required per covariate in ordinary, unweighted least-squares linear regression [6]. Whether this also applies to weighted random-effects meta-regression models is unclear. Given this uncertainty, we chose a cut-off of < 5 studies to identify meta-regression analyses at risk of overfitting. If a cut-off of < 10 studies had been used, as suggested in the Cochrane Handbook, the prevalence of meta-regression analyses at risk of overfitting would have been 53% (43 out of 81 meta-regression analyses, data not shown).

Meta-regression is often used to determine whether treatment effects are associated with the underlying baseline or population risk using the event rate observed in the control group as a surrogate for baseline risk [4, 9]. This approach is problematic as the observed risk included as a covariate is also incorporated in the expression of the treatment effect. Regression to the mean will therefore result in potentially spurious associations [1, 4, 8, 9]. Advanced methods should be used to overcome this problem [10–13].

Much like the decision to conduct a fixed-effect or random-effects meta-analysis, the decision to conduct a meta-regression analysis should not be based on heterogeneity assessments using, for instance, a chi-squared test, Cochrane Q, or I-squared [2]. Instead, a meta-regression analysis should be considered whenever a clinically important variation in treatment effects is observed on graphical display or indicated by the tau-squared [28].

## Conclusions

Results of the majority of meta-regression analyses based on aggregate data may be misleading. A considerable body of methodological literature and recommendations appear to have had little impact on the use of meta-regression in the medical literature. Authors, editors and peer reviewers need to become more aware of the methodological pitfalls of meta-regression analyses.

## Supplementary Information

**Additional file 1**

## Data Availability

The datasets used and analysed during the current study are available from the corresponding author on reasonable request.
