# Supplementary material for: Most published meta-regression analyses based on aggregate data suffer from methodological pitfalls: a meta-epidemiological study
Source: BMC Med Res Methodol. 2021 Jun 15;21:123. doi: 10.1186/s12874-021-01310-0 (PMC8207572; doi:10.1186/s12874-021-01310-0)
Supplement: Supplementary file 1 — Additional file 1 [file 12874_2021_1310_MOESM1_ESM.docx]

## **Appendix**

**Most published meta-regression analyses based on aggregate data suffer from methodological pitfalls: a meta-epidemiological study**

Michael Geissbühler^a,b†^, Cesar A. Hincapié^c,d,e†^, Soheila Aghlmandi^a,f^, Marcel Zwahlen^a^, Peter Jüni^e,g,h†^, Bruno R. da Costa^b,e,h†^

^a^ Institute of Social and Preventive Medicine (ISPM), University of Bern, Bern, Switzerland

^b^ Institute of Primary Health Care (BIHAM), University of Bern, Bern, Switzerland

^c^ Department of Chiropractic Medicine, Faculty of Medicine, Balgrist University Hospital and University of Zurich, Zurich, Switzerland

^d^ Epidemiology, Biostatistics and Prevention Institute (EBPI), University of Zurich, Zurich, Switzerland

^e^ Applied Health Research Centre (AHRC), Li Ka Shing Knowledge Institute of St. Michael’s Hospital, Toronto, Canada

^f^ Basel Institute for Clinical Epidemiology and Biostatistics, University Hospital Basel, Basel, Switzerland

^g^ Department of Medicine, University of Toronto, Toronto, Canada

^h^ Institute of Health Policy, Management and Evaluation (IHPME), Dalla Lana School of Public Health, University of Toronto, Toronto, Canada

Correspondence: Prof. Peter Jüni, email: [peter.juni@utoronto.ca](mailto:peter.juni@utoronto.ca)

^†^Michael Geissbühler and Cesar A. Hincapié contributed equally as first authors.

^†^Peter Jüni and Bruno R. da Costa contributed equally as last authors.

**Table A: Outcome variables**

| **Item** | **Definition** |
| --- | --- |
| Ecological fallacy (Pitfall 1) | We assessed if average patient characteristics have been related to the treatment effect in the meta-regression analysis. |
| Overfitting (Pitfall 2) | We assessed if the meta-regression analysis contains less than 5 component studies per covariate. |
| Meta-regression on risk of the analysed outcome (Pitfall 3) | We assessed if the outcome of the meta-regression analysis is an explicit function of the examined covariate.  In case of a binary outcome we considered meta-regression analyses relating the treatment effect to the rate of events in each study’s control group^1,2^ or an Abbé plot in conjunction with a regression analysis^1^ as potentially flawed due to regression to the mean. Another problematic analysis considered was a meta-regression model relating the treatment effect to average proportion of events in the treatment and control group^1^.  Similar problems are conceivable with continuous outcomes as well. First, if a continuous treatment effect is being related to the treatment effect in the control group, a regression to the mean bias can occur. Second, in a meta-regression analysis relating differences in change or final values to the baseline value of the same variable in each control group a regression to the mean bias can occur as well. Meta-regression analyses relating the average baseline value in the examined groups to the same continuous outcome variable were not considered as problematic, as this may be a small problem at most. |
| Discussion of the specific limitations | We assessed if the authors address the specific limitations of the assessed naïve meta-regression analyses and call the readers attention. |

**Table B: Characteristics of meta-regression analysis**

| **Item** | **Definition** |
| --- | --- |
| Outcome variable | We assessed whether the outcome variable (dependent variable) in the meta-regression model is binary, continuous or in case of multiple outcome variables also both. |

**Table C: Review characteristics**

| **Item** | **Definition** |
| --- | --- |
| **Journal characteristics** | |
| Name of the journal | Name of the journal as registered in the Medline database. |
| Journal Impact Factor (2007) | We took the Journal Impact Factor from the Journal Citation Reports Edition 2007 accessed through [www.webofknowledge.com](http://www.webofknowledge.com). We assumed any change in the journal impact factor to be gradual over the 10 year time period between 2002 and 2012, and so decided to use the 2007 impact factor—midpoint between 2002 and 2012—as an estimate of the average impact factor during the study period. With respect to reports published in journals that did not yet have an impact factor in 2007, we used the oldest available impact factor. Journals with no available impact factor were assigned an impact factor of 0. |
| Type of journal | We classified the journals appearing under the category “Medicine, general & internal” in the Journal Citation Reports accessed through [www.webofknowledge.com](http://www.webofknowledge.com) as general medical journals and all other journals as specialist medical journals. |
| Core clinical journal | Abridged Index Medicus (AIM or "Core Clinical") journal titles that were available online in PubMed as a search subset limit called "Core clinical journals” (<http://www.nlm.nih.gov/bsd/aim.html>, accessed on the 17^th^ of June 2014). |
| **Author characteristics** | |
| Affiliation to the industry | We considered any affiliation to the productive and non-productive industry of any listed article author. |
| Affiliation to an institute with statistical expertise | We considered the following type of institutes to have statistical expertise: clinical trials units, biostatistics, epidemiology, or public health institutes. If in doubt we consulted the internet for detailed information. |
| **Component studies** | |
| Number of included studies in the review | The overall number of clinical studies included in the review. |
| **Research field** | |
| Clinical field | We assessed the clinical field based on the condition of the patients studied. In the case of mixed conditions we took the therapy as a basis for the classification. Paediatrics was classified as such regardless of the patient’s condition. |
| Category of therapy | We classified the studies into dichotomised categories of the following types of therapy: drug, surgical, minimal invasive, device intervention, complex intervention, alternative, or other therapies. |

**Table D: Association between inappropriate meta-regression and review characteristics**

| **Ecological fallacy** | **Yes (n=53)** | **No (n=28)** | **Odds Ratio  (95% CI)** |
| --- | --- | --- | --- |
| Published in 2012 | 33 (62%) | 19 (68%) | 0.80 (0.31 to 2.06) |
| Journal characteristics |  |  |  |
| Core clinical journals | 11 (21%) | 8 (29%) | 0.65 (0.23 to 1.83) |
| General medical journals | 11 (21%) | 4 (14%) | 1.47 (0.44 to 4.88) |
| Impact factor higher than median | 26 (49%) | 14 (50%) | 0.96 (0.39 to 2.38) |
| Author characteristics |  |  |  |
| Affiliated with industry | 5 (9%) | 0 (0%) | 6.46 (0.34 to 121.27) |
| Affiliated with biostatistics or epidemiology department | 22 (42%) | 13 (46%) | 0.82 (0.33 to 2.03) |
| Ten or more of studies | 50 (94%) | 23 (82%) | 3.38 (0.81 to 14.06) |
| Drug intervention | 26 (49%) | 12 (43%) | 1.27 (0.51 to 3.15) |
| Binary outcome variable | 22 (42%) | 17 (61%) | 0.47 (0.19 to 1.18) |
| **Overfitting** | **Yes (n=14)** | **No (n=67)** | **Odds Ratio  (95% CI)** |
| Published in 2012 | 8 (57%) | 44 (66%) | 0.69 (0.22 to 2.15) |
| Journal characteristics |  |  |  |
| Core clinical journals | 3 (21%) | 16 (24%) | 0.95 (0.25 to 3.55) |
| General medical journals | 3 (21%) | 12 (18%) | 1.35 (0.35 to 5.18) |
| Impact factor higher than median | 7 (50%) | 33 (49%) | 1.03 (0.34 to 3.15) |
| Author characteristics |  |  |  |
| Affiliated with industry | 2 (14%) | 3 (4%) | 3.69 (0.65 to 20.84) |
| Affiliated with biostatistics or epidemiology department | 9 (64%) | 26 (39%) | 2.70 (0.85 to 8.61) |
| Ten or more of studies | 12 (86%) | 61 (91%) | 0.53 (0.11 to 2.57) |
| Drug intervention | 4 (29%) | 34 (51%) | 0.42 (0.13 to 1.38) |
| Binary outcome variable | 6 (43%) | 33 (49%) | 0.79 (0.26 to 2.43) |
| **Meta-regression on risk of the analysed outcome** | **Yes (n=5)** | **No (n=76)** | **Odds Ratio  (95% CI)** |
| Published in 2012 | 3 (60%) | 49 (64%) | 0.78 (0.14 to 4.21) |
| Journal characteristics |  |  |  |
| Core clinical journals | 2 (40%) | 17 (22%) | 2.43 (0.44 to 13.40) |
| General medical journals | 2 (40%) | 13 (17%) | 3.36 (0.60 to 18.88) |
| Impact factor higher than median | 3 (60%) | 37 (49%) | 1.47 (0.27 to 7.94) |
| Author characteristics |  |  |  |
| Affiliated with industry | 0 (0%) | 5 (7%) | 1.18 (0.06 to 24.27) |
| Affiliated with biostatistics or epidemiology department | 1 (20%) | 34 (45%) | 0.41 (0.06 to 2.75) |
| Ten or more of studies | 5 (100%) | 68 (89%) | 1.36 (0.07 to 26.91) |
| Drug intervention | 4 (80%) | 34 (45%) | 3.70 (0.55 to 24.75) |
| Binary outcome variable | 5 (100%) | 34 (45%) | 13.55 (0.72 to 253.7) |
| Odds ratios are for the comparison of meta-regression analyses with the characteristic as compared to meta-regression analyses without the characteristic. An odds ratio of 1.36 for ‘Ten or more studies’ indicates, for example, that the odds of meta-regression on risk of the analysed outcome is 1.36 times higher in meta-regression analyses that include 10 or more studies as compared with meta-regression analyses that include a lower number of studies. | | | |

**References**

1. Sharp SJ, Thompson SG, Altman DG. The relation between treatment benefit and underlying risk in meta-analysis. BMJ 1996;313(7059):735–8. https://doi.org/10.1136/bmj.313.7059.735

2. Thompson SG, Higgins JPT. How should meta-regression analyses be undertaken and interpreted? Stat Med 2002;21(11):1559–73. https://doi.org/10.1002/sim.1187

3. da Costa BR, Jüni P. Systematic reviews and meta-analyses of randomized trials: principles and pitfalls. European Heart Journal 2014;35(47):3336–45. https://doi.org/10.1093/eurheartj/ehu424
